# A Systematic Review of Marijuana Use and Outcomes in Patients with Myocardial Infarction

**DOI:** 10.7759/cureus.3333

**Published:** 2018-09-18

**Authors:** Ravi R Pradhan, Shashi R Pradhan, Shobha Mandal, Dhiri R Pradhan

**Affiliations:** 1 Internal Medicine, Tribhuvan University Teaching Hospital, Kathmandu, NPL; 2 Surgery, B.P. Koirala Institute of Health Sciences, Dharan, NPL; 3 Internal Medicine, Salem Internal Medicine PC, Pennsville, USA; 4 Internal Medicine, Yadukuha Primary Health Care Centre, Janakpur, NPL

**Keywords:** marijuana, legalization, prevalence, myocardial infarction, outcomes

## Abstract

The prevalence of marijuana use is increasing after its legalization in a few states of the United States (US). Smoking marijuana is found to be associated with an increased risk of myocardial infarction (MI) immediately after its use. However, knowledge about the impact of marijuana on outcomes following MI is limited. In light of the rapidly shifting landscape regarding the legalization of marijuana for medical and recreational purposes, it is necessary to evaluate the impact of marijuana on the outcomes following MI. In this systematic review, we opted to review the effects of marijuana on in-hospital and long-term outcomes following MI.

## Introduction and background

Laws and attitudes towards marijuana in the United States (US) are becoming more permissive. Marijuana is the most commonly used illicit drug in the US. The prevalence of marijuana use was 4.1% in 2001-2002 and 9.5% in 2012-2013 in the US [1]. After its legalization in a few states of the US, its use is becoming increasingly popular over time. Marijuana is found to be beneficial for the treatment of numerous health conditions such as cancer, glaucoma, Human Immunodeficiency Virus (HIV)/Acquired Immunodeficiency Syndrome (AIDS), and posttraumatic stress disorder [2]. The active constituent of marijuana is tetrahydrocannabinol, which is a mixed agonist for cannabinoid 1 and 2 receptors (CB1 and CB2) [3]. The activation of the CB1 receptor may increase lipid resistance and promote chronic cardiovascular dysfunction, particularly in obesity [4] and diabetes [5]. In contrast, the activation of CB2 receptors may suppress the inflammatory response and reduce atherosclerosis progression [6-7].

Marijuana use is found to be associated with adverse cardiovascular events. It increases sympathetic nervous system activity, which ultimately increases the heart rate, supine systolic, and diastolic blood pressures, leading to increased myocardial oxygen demand to a degree that the time to exercise-induced angina in patients with a history of stable angina may be decreased [8]. In addition, marijuana has been associated with triggering myocardial infarction (MI) in young male patients. After smoking marijuana, the risk of MI onset increases by 4.8 fold for the first 60 minutes. The annual risk of MI in a daily cannabis user increases from 1.5% to 3% per year [8].

In this systematic review, we reviewed the current evidence for the impact of marijuana use on outcomes following MI. The present work is, to our best knowledge, the most comprehensive systematic review of the latest four studies, allowing the direct comparison of the impact of marijuana on outcomes following cardiovascular incidents such as MI.

## Review

Methods

Search Strategy

**Figure 1:**
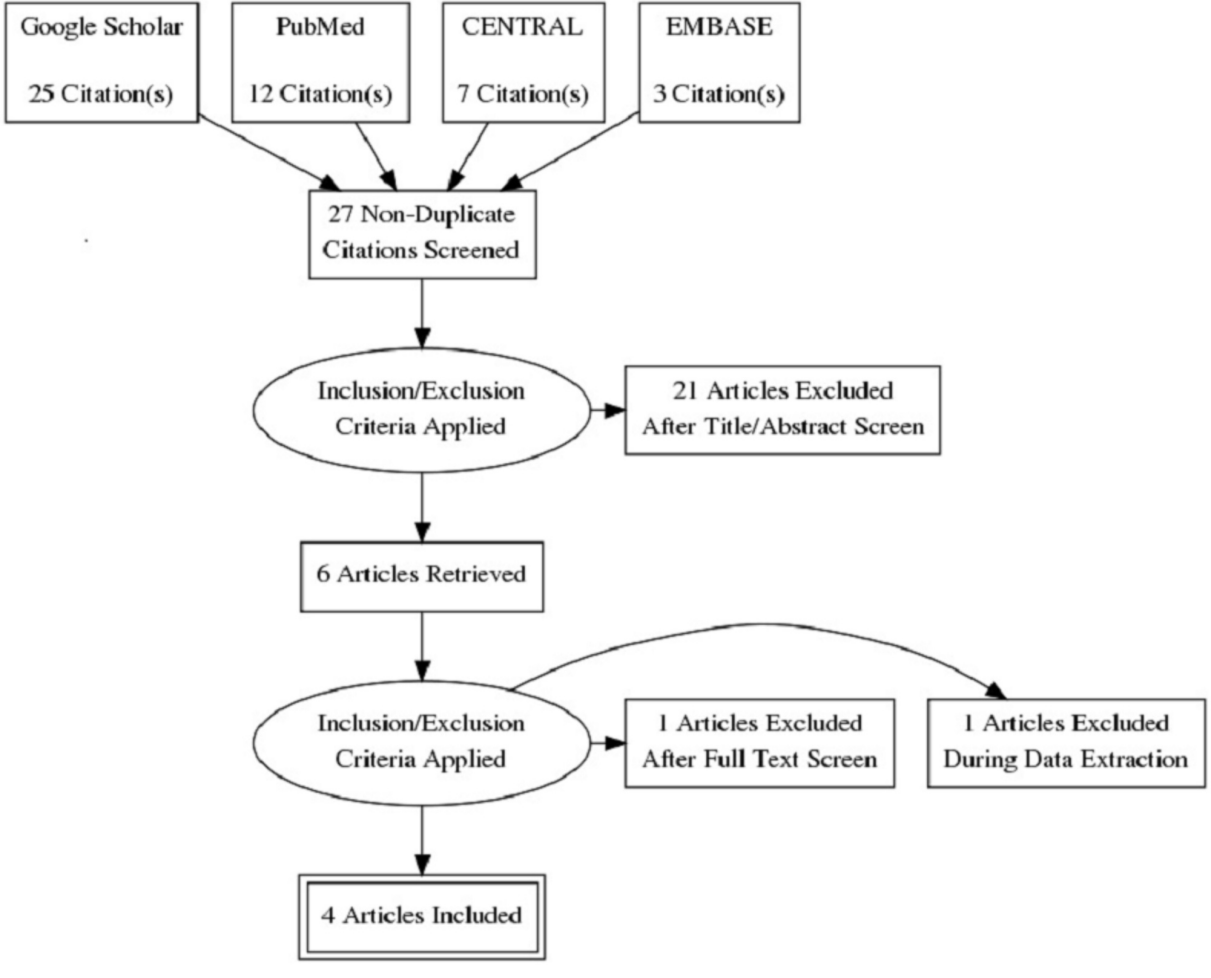
PRISMA diagram detailing the study identification and selection process. PRISMA: preferred reporting items for systematic reviews and meta-analyses

The PRISMA statement for reporting systematic reviews recommended by the Cochrane Collaboration was followed for conducting this systematic review (Figure 1). PubMed, Google Scholar, CENTRAL, and EMBASE were searched for peer-reviewed researches published between July 2001 and July 2018. Databases were searched using the search terms under two search themes and combined using the Boolean operator ‘AND’. For the theme ‘Marijuana’, we used text words: marijuana, cannabinoids, and tetrahydrocannabinol. For the theme ‘Myocardial Infarction', we used text words: myocardial infarction, acute myocardial infarction, ischemic heart disease, coronary artery disease, MI, AMI, IHD, and CAD.

Selection Criteria

Studies published in the English language were included in the review if they aimed to assess the impact of marijuana use on outcomes following MI. Studies that aimed to assess the impact of marijuana use on the outcomes of other diseases such as cancer, glaucoma, and posttraumatic stress disorder were excluded. In addition, case reports, editorials, and correspondences were also excluded. Diagram detailing the study identification and selection process is given in Figure 1.

Data Abstraction

The authors (RRP, SRP, and SM) screened the articles based on the inclusion and exclusion criteria. Full texts were obtained for articles that met the inclusion criteria. The authors developed a data abstraction spreadsheet using Microsoft Excel version 2013 (Microsoft Corp., Redmond, WA, USA) and included the following information: author, year of publication, journal, country where the study was done, study design, sample size, baseline characteristics of the patients, and outcomes in terms of morbidity and mortality. Any discrepancies were solved by consultation with the fourth author DRP.

Results and discussion

Study Characteristics

The study's characteristics are represented in Table 1. All the articles included in this review were of good quality, considering the presence of clear objectives, a clearly mentioned study design, and a clearly described statistical analysis. Four studies were included in this systematic review, with a total of 3,729,840 subjects. All the studies have used their own inclusion criteria. Studies conducted by Desai et al. [9] and Johnson-Sasso et al. [10] were retrospective, whereas Frost et al. [11] and Kenneth et al. [12] performed a cohort study, and they followed the patients for 18 and 3.8 years, respectively. All the studies were conducted in the USA.

**Table 1:** Key methodological characteristics of selected studies USA: United States of America; NA: not available; AMI: acute myocardial infarction; IABP: intraaortic balloon pump; STEMI: ST elevation myocardial infarction; NSTEMI: non-ST elevation myocardial infarction

Author	Year	Country	Journal	Sample size	Study design	Inclusion criteria	Primary outcome	Secondary outcome
Desai et al. [9]	2017	USA	Cureus	AMI without Marijuana: 2,416,162 AMI with Marijuana: 35,771	Retrospective	AMI patients aged 11 to 70 years	Prevalence of AMI; predictors of AMI incidence; inpatient mortality of AMI	Length of hospital stay; total hospital charges; complications of AMI
Frost et al. [11]	2013	USA	American Heart Journal	AMI without Marijuana: 1988 AMI with Marijuana: 109	Cohort study	Patients with creatine kinase level above the upper limit of normal, and positive MB isoenzymes; identifiable onset of symptoms of infarction; able to complete a structured interview	All-cause mortality; the association between marijuana use and the rate of mortality over up to 18 years of follow-up	NA
Johnson-Sasso et al. [10]	2018	USA	PLOS ONE	AMI without Marijuana: 1,270,043 AMI with Marijuana: 3,854	Retrospective	AMI patients aged >18 to <70 years	Composite of death; mechanical ventilation; cardiac arrest; placement of an intraaortic balloon pump (IABP); Shock	Individual components of the primary outcome; coronary angiogram; coronary percutaneous intervention; STEMI vs. NSTEMI
Kenneth et al. [12]	2008	USA	American Heart Journal	AMI without Marijuana: 1861 AMI with Marijuana: 52	Cohort study	Patients were required to have a creatine kinase level above the upper limit of normal; positive MB isoenzymes; identifiable onset of symptoms of infarction; ability to complete a structured interview	All-cause mortality	Cardiovascular and noncardiovascular mortality

Patient Characteristics

The patient characteristics of the study are shown in Table 2. In all the studies, marijuana users tended to be younger, male, and current smokers and had less co-morbidity than the non-users. The data about the mean body mass index (BMI) was available only in two studies [11-12]. The mean BMI (Kg/m^2^) values reported in the study by Frost et al. [11] and Kenneth et al. [12] were 28.3 ± 5.2 and 27.3 ± 5.2 in the acute myocardial infarction (AMI) without marijuana group and 29.9 ± 5.6 and 27.8 ± 5.3 in the AMI with marijuana group, respectively. 

**Table 2:** Baseline characteristics of patients included in selected studies SD: standard deviation; BMI: body mass index; AMI: acute myocardial infarction; NA: not available

Study	Mean age ± SD	Male (%)	BMI (kg/m^2^)	Alcohol Abuse (%)	Smoking (%)	Cocaine Abuse (%)	Diabetes (%)	Hypertension (%)	Dyslipidemia (%)
Desai et al. [9]	AMI without Marijuana: 57.79 ± 8.98	AMI without Marijuana: 66.0	AMI without Marijuana: NA	AMI without Marijuana: 5.1	AMI without Marijuana: 46.3	AMI without Marijuana: 1.2	AMI without Marijuana: 30.0	AMI without Marijuana: 67.6	AMI without Marijuana: 58.9
	AMI with Marijuana: 49.34 ± 10.80	AMI with Marijuana: 76.9	AMI with Marijuana: NA	AMI with Marijuana: 22.6	AMI with Marijuana: 75.9	AMI with Marijuana: 18.9	AMI with Marijuana: 18.3	AMI with Marijuana: 58.9	AMI with Marijuana: 50.6
Frost et al. [11]	AMI without Marijuana: 52.3 ± 7.7	AMI without Marijuana: 77	AMI without Marijuana: 28.3 ± 5.2	AMI without Marijuana: NA	AMI without Marijuana: 48	AMI without Marijuana: 1	AMI without Marijuana: 17	AMI without Marijuana: 37	AMI without Marijuana: NA
	AMI with Marijuana: 43.7 ± 8.2	AMI with Marijuana: 93	AMI with Marijuana: 29.9 ± 5.6	AMI with Marijuana: NA	AMI with Marijuana: 67	AMI with Marijuana: 16	AMI with Marijuana: 9	AMI with Marijuana: 28	AMI with Marijuana: NA
Johnson-Sasso et al. [10]	AMI without Marijuana: 57.2	AMI without Marijuana: 66	AMI without Marijuana: NA	AMI without Marijuana: NA	AMI without Marijuana: 27	AMI without Marijuana: NA	AMI without Marijuana: 32	AMI without Marijuana: 57	AMI without Marijuana: 41
	AMI with Marijuana: 47.2	AMI with Marijuana: 76	AMI with Marijuana: NA	AMI with Marijuana: NA	AMI with Marijuana: 59	AMI with Marijuana: NA	AMI with Marijuana: 19	AMI with Marijuana: 53	AMI with Marijuana: 43
Kenneth et al. [12]	AMI without Marijuana: 62.0 ± 12.3	AMI without Marijuana: 68	AMI without Marijuana: 27.3 ±5.2	AMI without Marijuana: NA	AMI without Marijuana: 32	AMI without Marijuana: NA	AMI without Marijuana: 21	AMI without Marijuana: 45	AMI without Marijuana: NA
	AMI with Marijuana: 42.6 ± 8.8	AMI with Marijuana: 94	AMI with Marijuana: 27.8 ±5.3	AMI with Marijuana: NA	AMI with Marijuana: 77	AMI with Marijuana: NA	AMI with Marijuana: 8	AMI with Marijuana: 23	AMI with Marijuana: NA

Outcomes

Desai et al. [9] evaluated that the odds of all-cause in-hospital mortality were not significantly increased in the AMI with marijuana group as compared to the AMI without marijuana group when adjusted for age, race, the length of stay, the median house of income in the zip code, an indicator of sex, hospital bed size, smoking, and cocaine abuse (adjusted OR: 0.742, CI: 0.693-0.795, *p *< 0.001). The mean length of stay (in days; 4.7 ± 5.9 vs. 5.6 ± 8.0) and the total hospital charges ($76,272.23 vs. $85,702.22) were lower in the AMI with marijuana group (*p *< 0.001).

A study conducted by Frost et al. [11] found that compared to the marijuana nonusers, the mortality rate was 29% higher among the marijuana users, but this did not reach statistical significance (95% CI, 0.81-2.05, *p* = 0.28).

In the study of Johnson-Sasso et al. [10], there was no association between marijuana use and the primary outcome (*p* = 0.53). The in-hospital mortality rate was significantly lower in the marijuana users compared to the nonusers (OR: 0.79, *p* = 0.016). Apart from the mortality benefit, marijuana users were significantly less likely to experience shock (OR: 0.74, *p* = 0.001), or require an intraaortic balloon pump placement (IABP; OR: 0.80, *p* = 0.03) following AMI. However, marijuana users were more likely to be placed on mechanical ventilation (OR: 1.19, *p* = 0.004).

Kenneth et al. [12] discovered that marijuana users had three-fold higher mortality in comparison to the nonusers after being adjusted for age and sex, and in fully adjusted models. Compared with the nonusers, the hazard ratios for marijuana usage less than weekly and weekly or more were 2.5 (95% CI, 0.9-7.3), and 4.2 (95% CI, 1.2-14.3), respectively. For cardiovascular mortality and non-cardiovascular mortality, the age- and sex-adjusted hazard ratios with any use were 1.9 (95% CI, 0.6-6.3), and 4.9 (95% CI, 1.6-14.7), respectively. In the same study, while comparing 42 marijuana users and 42 other patients matched on propensity scores, there were six deaths among marijuana users and one among non-users (log-rank *p* = 0.06).

Besides, in a study conducted by Vin-Raviv et al. [13] among cancer patients (*n *= 387,608), odds of in-hospital mortality was found to be significantly reduced among marijuana users compared with non-users (OR: 0.44, 95% CI: 0.35-0.55). In the same study, they also established that marijuana use was associated with significantly reduced odds of heart failure and cardiac disease compared with non‐users. 

Discussion

Marijuana is becoming increasingly available to the general population after its legalization in several states of the USA. As both medical and recreational use of marijuana is increasing, the knowledge and attitude among healthcare workers and patients about marijuana are important. Both healthcare providers and patients must carefully balance the anticipated benefits and the established health risks. 

In our review, two out of four studies are cohort studies, and both studies found that marijuana use was associated with higher folds of mortality. It is in contrast with two retrospective studies that demonstrated decreased all-cause mortality following MI. Since retrospective studies cannot establish a causal relationship, at this point, we cannot conclude if marijuana is beneficial or harmful, which remains a major limitation of our review. Nevertheless, the results of our review should not be neglected. Further large cohort study with sufficient follow-up time is required to establish the causal relationship between marijuana use and the outcomes following MI. Additionally, clinical trials on supplementation of medical marijuana in MI patients can be done to observe the direct effect. But conducting a randomized controlled trial (RCT) by supplementing medical marijuana will raise an ethical issue. Hence, RCT seems to be impractical at this point in time. 

The major limitations of the included studies are represented in Table 3. All the studies have included the patients with MI who were admitted to the hospital. Hence, the possibility of unmeasured or residual confounding was not ruled out in all four studies. In the retrospective studies of Desai et al. [9] and Johnson-Sasso et al. [10], patients were not followed up to evaluate the long-term outcomes of marijuana in MI patients.

**Table 3:** Major limitations of the study MI: Myocardial infarction

Study	Major limitations
Desai et al. [9]	Only studied in-hospital odds of mortality, which leaves out outpatients or post-discharge odds of mortality in MI patients
Frost et al. [11]	Cannot rule out the possibility of unmeasured or residual confounding; because most of the patients with MI were on some medications before sustaining MI, so they might have received secondary prevention measures in a manner unrelated to marijuana use; the study was based on self-reported marijuana use, so there may be some exposure misclassification
Johnson-Sasso et al. [10]	Angiograms, laboratory tests, medications taken pre- or post-MI, and vital signs on admission were not available; no post-discharge data including long-term mortality and readmissions; the route, amount and frequency of marijuana use in each patient could not be determined, so a dose-response effect could not be established
Kenneth et al. [12]	The number of marijuana smokers was relatively small; follow-up was limited to approximately 4 years; could not prove cause and effect relationship

The present work is, to our best knowledge, the most comprehensive systematic review of the latest four studies, allowing a direct review of marijuana use and outcomes in patients with MI. Despite a relatively small number of the available original studies, the number of patients included in these studies was large.

## Conclusions

To conclude, this review has found that in-hospital mortality in patients with MI was significantly reduced among marijuana users compared with non-users in retrospective studies but not in cohort studies. However, we could not conclude whether the outcomes of retrospective studies occurred due to a direct causal relationship or by chance. Therefore, additional large cohort studies and clinical trials are required to establish the relationship.
